# Responses of Diversity and Productivity to Organo-Mineral Fertilizer Inputs in a High-Natural-Value Grassland, Transylvanian Plain, Romania

**DOI:** 10.3390/plants11151975

**Published:** 2022-07-29

**Authors:** Ioan Gaga, Florin Pacurar, Ioana Vaida, Anca Plesa, Ioan Rotar

**Affiliations:** Department of Grasslands and Forage Crops, Faculty of Agriculture, University of Agricultural Sciences and Veterinary Medicine Cluj-Napoca, Calea Manastur 3-5, 400372 Cluj-Napoca, Romania; ioan.gaga@yahoo.com (I.G.); anca.plesa@usamvcluj.ro (A.P.); ioan.rotar@usamvcluj.ro (I.R.)

**Keywords:** high-nature-value grassland (HNV), indicator species, Transylvanian Plateau, grassland biodiversity, organic and mineral fertilization, *Festuca rupicola*, grassland management

## Abstract

Ecosystems with high natural value (HNV) have generally been maintained by agricultural practices and are increasingly important for the ecosystem services that they provide and for their socio-economic impact in the ever-changing context. Biodiversity conservation is one of the main objectives of the European Green Deal, which aims to address biodiversity loss, including the potential extinction of one million species. The aim of this research was to trace the effects of organic and mineral fertilizers on the floristic composition, but also on the number of species, of the grasslands with high biodiversity (HNV) from the Transylvanian Plain, Romania. The experiments were established in 2018 on the nemoral area and analyzed the effect of a gradient of five organic and mineral treatments. Fertilization with 10 t ha^−1^ manure or N_50_ P_25_K_25_ ensures an increase in yield and has a small influence on diversity, and it could be a potential strategy for the maintenance and sustainable use of HNV grasslands. Each fertilization treatment determined species with indicator value that are very useful in the identification and management of HNV grasslands. The dry matter biomass increases proportionally as the amounts of fertilizer applied increase and the number of species decreases.

## 1. Introduction

In Europe, semi-natural grasslands with extensive management are considered sources of biodiversity, some of which are even of major international importance, competing with the diversity of habitats that have world records in the number of species per unit area [1,2]. These ecosystems, with high natural value (HNV), have generally been maintained by agricultural practices, so their phytodiversity has developed over the centuries in close correlation with the type of management applied [3,4,5]. Semi-natural grasslands, mainly used for feed production, are an important component of the use categories in Europe, covering more than a third of the agricultural area [6,7]. The biodiversity and especially the floristic diversity of semi-natural grasslands have become a major concept in agronomic research. The high diversity of semi-natural grasslands brings many benefits to farmers and consumers, as well as ecosystem services. These include, in the agronomic context, the harvest, the source of nutrients through the decomposition of organic matter, the cessation of nutrient washing, pollination, soil conservation, and resistance to invasive species in the face of climate change [8,9]. Decreased biodiversity can affect the functions and services of ecosystems [10]. Moreover, in order to maintain a characteristic floristic composition, proper management is needed, with reduced quantities of fertilizers [11]. There is a large amount of research that has focused on increasing the production of grasslands and has looked at the effect of applying organo-mineral fertilizers, both in terms of productivity and floristic composition [2,12]. In terms of grassland productivity at the European level, it plays an important role as a source of fodder for both domestic and wild animals. At the same time, grasslands have a multifunctional ecological role, forming an ecosystem that is a habitat for flora and fauna [13]. The growing need for food due to the increase in population has led to the significant intensification of grassland areas, especially in the last 50 years [14]. The development of evaluation methods and the establishment of indicators to highlight the effect of management on grassland systems is a current concern [15,16]. The effect of management on the productivity and biodiversity of high-natural-value pastures (HNV) is increasingly being addressed at the European level [17]. Currently, in Romania, specialists are trying to assess the management of high-biodiversity (HNV) grasslands with the help of species with indicator value and to draw up a list of species taking into account the stationary conditions and the intensity of the management used [2,11,17,18]. An innovative feature of this research is the analysis of the reaction of semi-natural grasslands to treatment with organic and mineral fertilizers. In order to identify the ecological and agronomic value of semi-natural grasslands, both knowledge on the ecology of plant communities and knowledge on indicator plant species are needed [19,20]. Until now, research has analyzed the effect of fertilizers on the yield of dry matter (DM) and, later, the study of the floristic composition. It was stated that the crop of dry matter increases as a result of the increase in the quantities of fertilizer but did not correlate with the new types of grasslands installed, as a result of intensification, and increased productive potential was noted. Therefore, the present research first proposes an analysis of the reaction of the floristic composition (at the applied inputs) and then an analysis of the dry matter crop (DM). At the same time, the current threats to these systems are diverse and persist, despite global and European (*Green Deal*) policies that address these shortcomings. Climate change, abandonment, and intensification of systems are just a few of them. The sustainability of the use of natural and semi-natural grassland systems is current and widely debated, from the global to the regional level, in many areas of activity [21].

The aim of this research was to trace the effects of organic and mineral fertilizers on the floristic composition, but also on the biodiversity, of the HNV grasslands on the Transylvanian Plain. The aim of our research is influenced by the current context of the European Green Pact. The uniqueness of this study shows that, regarding the science of grassland ecosystems, the application of fertilizers is applied following economic criteria to increase productivity. Until now, research has analyzed the effect of fertilizers on biomass (D.M) and later the floristic composition, a situation that provided an incomplete information. Over time, this mode of analysis can lead to a restriction of the number of species in grassland ecosystems. In most studies it was stated that biomass increases as a result of increasing amounts of fertilizers, but does not correlate with new types of grass installed, as a result of intensification and which had an increased productive potential. Therefore, this paper first proposes a treatment of the reaction of the floristic composition (at the applied inputs) and then an analysis of the biomass. One solution that could be beneficial for grassland ecosystems would be to install short-term experiences, experiences that give us a quick forecast of the changes that occur in these grassland ecosystems. Vegetation analysis was performed both quantitatively and qualitatively. The objectives evaluated in the experiment were formulated in the form of questions: (i) With which amount of fertilizer are there major changes in the structure of the floristic composition? (ii) What is the amount of fertilizer until which the phytocenosis does not lose its biodiversity? (iii) Can plant species with indicative value for applied management be identified? (iv) Which optimal fertilizer doses can be identified so that there is a balance between productivity and biodiversity?

## 2. Results

### 2.1. The Influence of Mineral and Organic Fertilizers on the Floristic Composition

**Figure 1:**
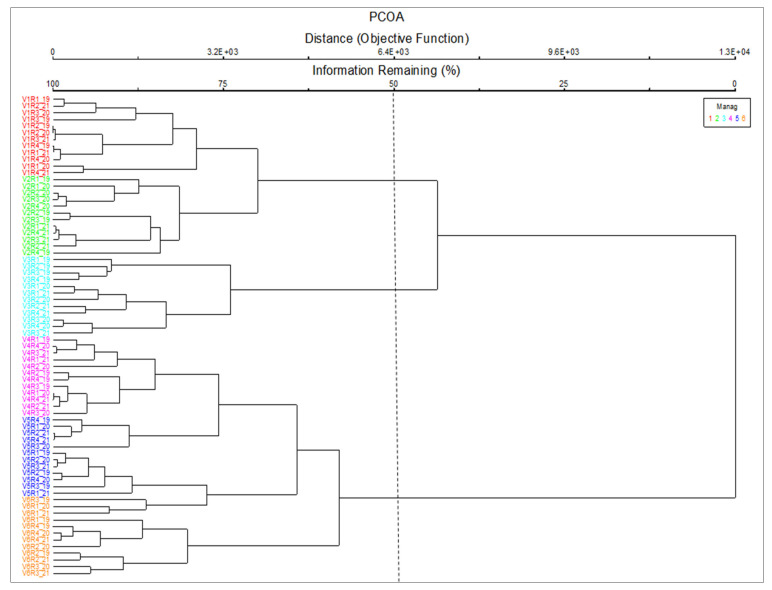
Dendrogram of floristic plot classification. V1—natural grasslands (control); V2—10 t/ha^−1^ manure; V3—10 t ha^−1^ manure + N_50_ P_25_K_25_; V4—N_50_ P_25_K_25_; V5—N_100_ P_50_K_50_; V6—10/t ha^−1^ manure + N_100_ P_50_K_50_; R1—repetition 1; R2—repetition 2; R3—repetition 3; R4—repetition 4, etc. 19—year 2019; 20—year 2020; 21—year 2021; V1R1_19—natural grasslands (control), repetition 1, year 2019; V1R2_19—natural grasslands (control), repetition 2, year 2019, etc.; V2R1_19—10 t/ha^−1^ manure, repetition 1, year 2019; V2R2_19—10 t/ha^−1^ manure, repetition 2, year 2019, etc.; V3R1_19—10 t ha^−1^ manure + N_50_ P_25_K_25_ repetition 1, year 2019; V3R2_19—10 t ha^−1^ manure + N_50_ P_25_K_25_ repetition 2, year 2019, etc.

Based on the cluster analysis, it is possible to observe the classification of the vegetation and the modification of the type of grassland due to the floristic distances between them (Figure 1). The cut-off level of the dendrogram was established on the basis of phytosociological and ecological meaning, in order to include as much information as possible [22]. Based on the analysis of the floristic composition, we considered that cutting off at the value of 50 is an optimal solution, this having the highest phytosociological, ecological, and agronomic meaning. Thus, three distinct groups were identified. The formation of groups because of the application of inputs shows that fertilizer treatments have produced major changes in the vegetation. Each amount of fertilizer applied determined a particular floristic composition. The first group is represented by V1 and V2. The second group is represented by V3, and the last group by the V4, V5, and V6 variants. In V1, we have the type of *F. rupicola* grassland. In variants V2, V3, and V4, there are changes inside the phytocenosis, but these are not major, because the type of grassland which remains is *F. rupicola*. Changes in the type of grassland occur in V5 and V6 when we have the grassland *A. capillaris* with *F. rupicola*.

Axis 1 is represented as a proportion of 78.8%, and axis 2 of 11.9% (Table 1).

**Figure 2:**
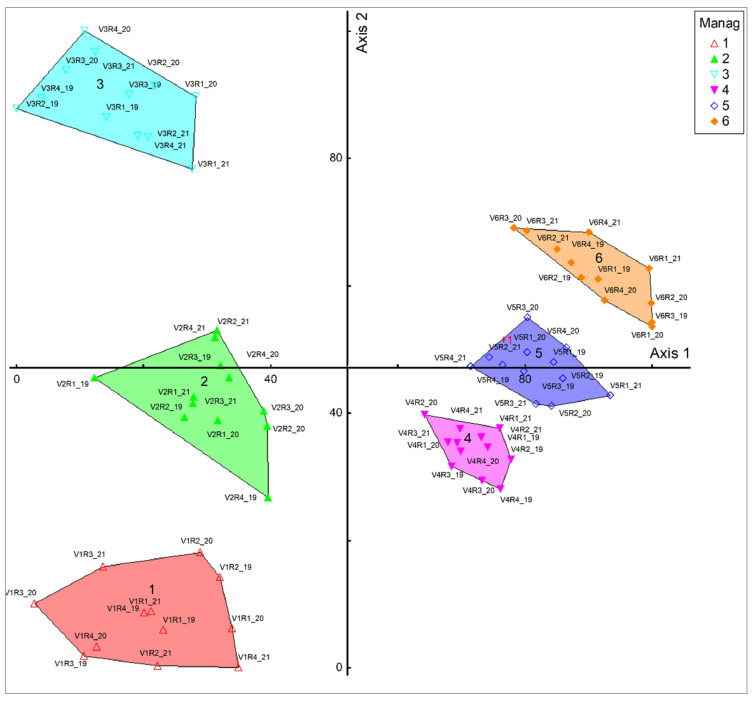
Influence of treatments on the floristic composition. V1—natural grasslands (control); V2—10 t/ha^−1^ manure; V3—10 t ha^−1^ manure + N_50_ P_25_K_25_; V4—N_50_ P_25_K_25_; V5—N_100_ P_50_K_50_; V6—10t ha^−1^ manure + N_100_ P_50_K_50_; R—repetition; 19—year 2019; 20—year 2020; 21—year 2021; Group 1 = V1; 2 = V2; 3 = V3; 4 = V4; 5 = V5; 6 = V6.

Following the orders with the PCOA resulted in Figure 2. The control phytocenosis is represented by the type of *F. rupicola*; in variants V2, V3, and V4, 2 years after the application, there were only changes inside the phytocenosis, with no changes in the type of grassland. In fact, a significant change in the level of the vegetal cover occurs when the quantity of fertilizer increases—namely, for the application with N_100_P_50_K_50_ kg (V5), respectively, and the application of the combination of mineral and organic fertilizers (V6). When applying these treatments, *A. capillaris* grassland with *F. rupicola* is installed. 

Following the analysis of the floristic composition for the years 2019, 2020, and 2021, we obtained the following results. Some plant species (31 plant species) correlate with axis 1 of sorting and others (21 plant species) correlate with axis 2 of sorting, which means that they are favored by the absence of fertilization or by fertilization with small quantities of fertilizer (Table 2). Among them, we mention *F. rupicola* (*p* < 0.001), *Lolium perenne*, *Vicia cracca*, *Achillea millefolium*, *Plantago media*, etc. For these plant species, the application of 10 t/ha^−1^ manure annually leads to an improvement in the soil nutrients, so these plant species are no longer found in the ecological optimum. Some of the plant species have their ecological optimum between the treatment with 10 t/ha^−1^ manure and the non-application of fertilization, such as *Plantago lanceolata* (*p* < 0.001), *Onobrychis viciifolia* (*p* < 0.001), and *Bromus secalinus* (*p* < 0.001).

The application of manure (V2) has led to the extinction of some plant species and the appearance of others. In particular, the application of 10 t ha^−1^ manure (V2) annually determined the disappearance from the floristic composition of the following species: *Agropyron intermedium*, *Brachypodium pinnatum*, *Carex humilis*, *Carthamus lanatus*, *Bupleurum falcatum*, *Allium angulosum*, *Nigella arvensis*, and *Scabiosa ochroleuca*. Moreover, it caused the appearance of nine new plant species in the floristic composition: *Dactylis glomerata*, L. *perenne*, *Trifolium pratense*, *Trifolium repens V. cracca*, etc. With the application of 10 t/ha^−1^ manure + N_50_ P_25_ K_25_ (V3), three plant species disappeared from the phytocenosis (*Coronilla varia*, *Cerastium holosteoides*, *Poa angustifolia*), with the appearance of ten plant species in the floristic composition, such as *Festuca arundinacea*, *Festuca pratensis*, *Poa pratensis*, *B. secalinus*, *C. humilis*, *Medicago sativa*, *Salvia pratensis*, and *Centaurea stoebe*. This analysis was performed in comparison to V1. The variant V4 with N_50_ P_25_ K_25_ led to the extinction of three plant species (*B. pinnatum*, *Eryngium campestre*, and *C. varia)* and the emergence of four new species (*Arrhenatherum elatius*, *F. arundinacea*, etc.). Treatment (V5) caused the plant species *Bromus inermis* to appear and led to the extinction of the species *O. viciifolia* from the floristic composition of the grassland type. Regarding the last degree of intensification of the phytocenosis, the application of 10 tha^−1^ manure + N_100_ P_50_K_50_ (V6) determined the emergence of mesotrophic and eutrophic plant species, species that find their ecological optimum at this degree of fertilization. In the case of this treatment, the floristic composition is restricted, so 10 plant species disappeared from the floristic structure: *P. lanceolata*, *Elymus repens*, *Fragaria viridis*, *Convolvulus arvensis*, etc.

### 2.2. Effects of Fertilization on Grassland Biodiversity (Number of Species)

**Figure 3:**
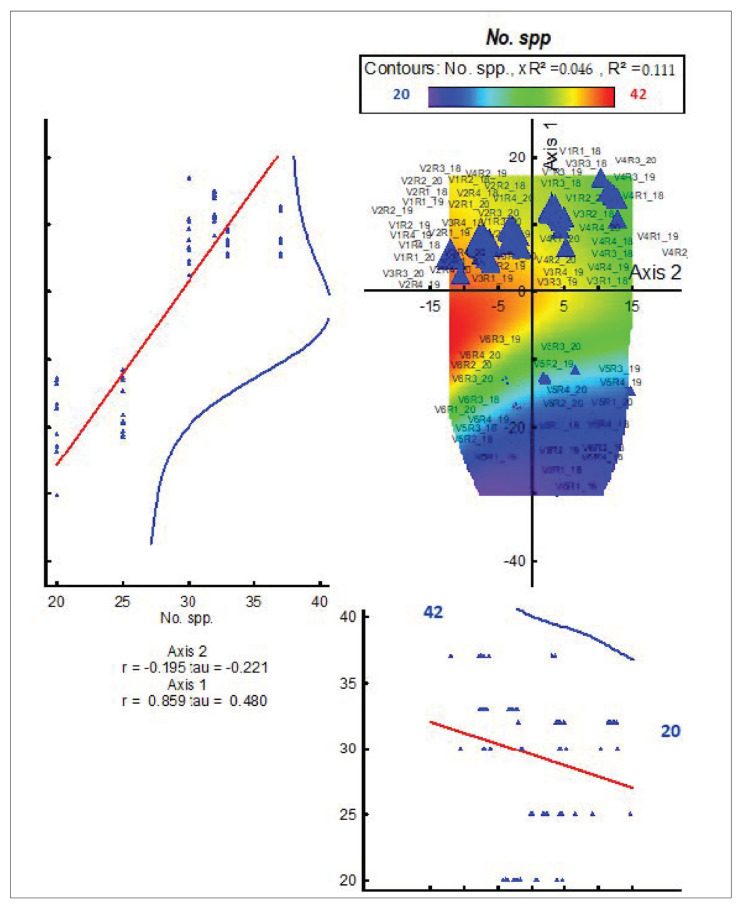
Influence of treatments applied on grassland biodiversity (number of plant species). r—Pearson correlation coefficient; tau—rank (Kendall’s tau) relationships between the ordination scores and the individual variables; V1—semi-natural grassland (control); V2—10 t/ha^−1^ manure; V3—10 t ha^−1^ manure + N_50_ P_25_K_25_; V4—N_50_ P_25_K_25_; V5—N_100_ P_50_K_50_; V6—10 t ha^−1^ manure + N_100_ P_50_K_50_. R—repetition; 19—year 2019; 20—year 2020; 21—year 2021; No. Spp.—number of plant species; 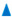
—experimental variant; the red line represents the regression (species number trends), and the blue line is represented by the maximum amplitude curves; left graph is representing the axis 2 and the bottom graph is representing the axis 1.

In Figure 3, axis 1 has the greatest importance in explaining the phenomenon, namely r = 0.859, tau = 0.480 (Figure 3). The high number of plant species is related to the type of fertilizer applied, but especially to the dose administered. In our experience, the biodiversity of the grassland has suffered, as it has decreased from 42 plant species in the phytocenosis of the control to 20 plant species in the phytocenosis where the most intensive management measures have been applied, namely in V6 (Axis 1 = 0.895, Figure 3). 

In the control phytocenosis, we identified a total of 42 species. When applying the quantity of 10 t ha^−1^ manure, small changes were registered at the level of the floristic composition. In this phytocenosis (V2), we identified 39 plant species, which were registered in the floristic composition. Therefore, three plant species have disappeared from the control (V1) phytocenosis. When applying treatment three (V3), in the floristic composition, we identified 36 plant species. Compared to the control variant, it was observed that there was an important change in the floristic composition: six plant species had disappeared from the phytocenosis. The application of mineral fertilizers strongly influences the participation of plant species, causing the disappearance or appearance of new species, compared to the application of organic inputs. The application of N_50_P_25_K_25_ (V4) led, in the floristic composition, to 35 plant species. A significant change in the floristic composition was registered when applying the quantities of N_100_P_50_K_50_ (V5), the number of plant species being significant, only identifying 25 species of plants. In the case of this treatment, a loss of biodiversity can be observed in the grasslands with HNV in the study area. The increase in the fertilization quantities and the application of combined fertilization of (V6) caused a drastic decrease in the number of plant species: in the floristic composition, there are only 20 species of plants. Compared to the control variant, a loss of biodiversity can be noticed for the grasslands with HNV in the study area (Figure 3).

### 2.3. Species with Indicative Value for the Intensity of Applied Management

One of the objectives of this research was to identify plant species with indicator value for each graduation of fertilization applied, for the type of fertilizer applied, and for organic or mineral fertilization. The phytocenosis of the control (V1) had 36 species of plants. The absence of fertilizer inputs was highlighted in many plant species, with most species having a very significant indicator value. Thus, some plant species with the highest indicator value have been identified (100.0) as follows: *A. intermedium*, *A. elatius*, *F. arundinacea*, *B. inermis*, *B. secalinus*, *C. stoebe*, *C. lanatus*, *B. falcatum*, *A. angulosum*, *N. arvensis*, and *S. ochroleuca*. In the control phytocenosis (T1), we identified 12 plant species with indicator value (*Festuca valesiaca*, *B. pinnatum*, *C. stoebe*, *Tragopogon dubius*, *P. angustifolia*, etc.). In the phytocenosis where we applied 10 t ha^−1^ manure (T2), in the floristic composition, there were five indicator plant species (*E. repens*, *T. repens*, *V. cracca*, etc.). Treatment (T3) revealed nine species of plants with indicative value in the floristic composition (*A. elatius*, *F. arundinacea*, *B. secalinus*, *Agrimonia eupatoria*, etc.). Regarding T4 with N_50_ P_25_ K_25_, it revealed only one indicator plant species (*Poa pratensis*). T5 had, in the floristic composition, eight species with indicator value (including *F. rupicola*, *Festuca pratensis*, and *P. lanceolata Galium verum*); in treatment T6, we identified in the floristic composition a total of seven plant species with indicator value (*A. capillaris*, *Dactylis glomerata*, *L. perenne*, *Lotus corniculatus*, *T. pratense*, etc., Table 3). The indicator species listed above provide us with valuable information on the management applied in these HNV systems. Once the phytocenosis situation has been established, appropriate practical management strategies can be developed for the future, including measures for maintenance and use. The elaboration of this list of species with indicative value (Table 3) for the degree of intensity of organic, mineral, and combined fertilization (organo-mineral) is very beneficial because, in the near future, the evaluation of the grasslands will be carried out according to the result. The list of species with indicator value developed in this paper ensures support for the beneficiaries of environmental and climate measures, in order to self-assess practices on the farm, as well as to support officials within the institutions involved in verifying compliance with commitments. 

### 2.4. The Influence of Organic Fertilizer Gradient over Agronomic Spectrum

**Figure 4:**
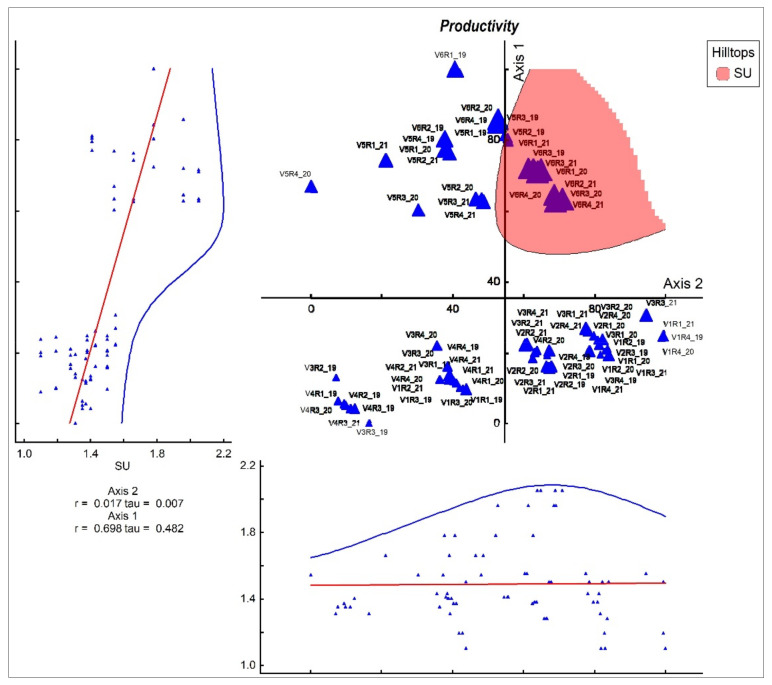
Influence of treatments applied on agronomic factors (DM—biomass); r—Pearson correlation coefficient; tau—rank (Kendall’s tau) relationships between the ordination scores and the individual variables; V1—semi-natural grasslands (control); V2—10 t ha^−1^ manure; V3—10 t ha^−1^ manure+ N_50_ P_25_K_25_; V4—N_50_ P_25_K_25_; V5—N_100_ P_50_K_50_; V6—10 t ha^−1^ manure+ N_100_ P_50_K_50_. R—repetition; 19—the year 2019; 20—the year 2020; 21—the year 2021; SU—dry matter biomass; 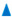
—experimental variant; the red line represents the regression and the blue line is represented by the maximum amplitude curves; left graph is representing the axis 2 and bottom graph is representing the axis 1.

The dry matter biomass increases proportionally as the amounts of fertilizer applied increase. The amount of biomass correlates significantly (r = 0.698; Figure 4) with the applied treatments, but especially with those applied in variant 6. The productivity of *F. rupicola* grasslands (control) is 1.19 t ha^−1^ (DM), and after the application of the treatments, it increases up to 2.05 t ha^−1^. In our experience, the difference in biomass between the control variant and the application of the treatment with 10 t ha^−1^ manure brought about significant increases in dry matter (0.25 t ha DM). Increasing the amount of fertilizer registered higher production increases, but at the same time, it decreased the biodiversity of the grasslands with HNV. Thus, the application of organic fertilizers in moderate doses of 10 t ha^−1^ manure registers a significant increase in biomass, but at the same time, there is a reduction in the diversity of grasslands with HNV. Consequently, the application of mineral fertilizers in doses of N_50_P_25_K_25_ does not produce an imbalance in phytocenosis, registering an increase in biomass but with a minimal decrease in the number of plant species in the floristic composition (Figure 4).

## 3. Discussion

The application of fertilizers on semi-natural HNV grasslands determines a clear classification of phytocenoses. The floristic composition of a grassland is a reflection of the phytocoenosis and the practical management applied [8,23]. Each phytocenosis, with its own characteristics, can be influenced by humans and, therefore, new types of grasslands appear [2]. Organized experiments, both nationally and internationally, have shown that the intensification of grassland systems greatly reduces the specific richness, installing valuable forage species (generally nitrophilic species), which offer rich biomass crops and high-quality fodder [13,17,24,25,26]. The formation of specific groups as a result of the application of inputs demonstrates that fertilizer treatments have produced major changes in the phytocenosis of grasslands [27]. Moreover, in a study conducted in the period 2002–2003, on the indicator species in various types of grasslands in the alpine area of Austria, it was specified that the applied management was the one that ensured the classification of floristic plots according to the similarity of the floristic composition of the types of grasslands analyzed. Although the study focused on the chemical properties of the soil and the substrate of the floristic cover, the author noted a clear relationship between the intensity of grassland management and the diversity of the floristic plots [28,29,30]. In a study carried out on permanent grasslands in the southern part of Tyrol (Italy), the authors observed the positive effects of applying organic fertilizers in moderate doses on the species *T. pratense*, in less dry years; however, researchers have expressed concern regarding the recurrence of drought and the complexity of applying fertilizers, noting that this complexity negatively affects the floristic composition and biodiversity of permanent grasslands [31]. In our research, the species *T. pratense* is recommended for treatment with 10 t/ha manure^−1^ + N_100_ P_50_K_50_ (V6).

It is demonstrated in the literature that the species *A. capillaris* increases its coverage in the vegetal cover as the fertilizer dose increases [32]. Thus, our results are also supported by specialized research, where, in a similar experience but on another type of grassland, namely *Festuca rubra*, the species *A. capillaris* was strongly influenced by the treatments applied and had the highest coverage after treatments with N_100_P_50_K_50_ and N_150_P_75_K_75_, with the plant species increasing its share from 12.5% coverage (control) to 62.5% in the floristic composition after treatment with N_150_P_75_K_75_ [2,4,18,33]. 

The richness of the number of plant species is determined by the type of fertilizer applied, but especially by the dose of fertilizer administered [34]. Numerous studies have shown a positive relationship between biodiversity and low-dose fertilization [7,35,36]. A study similar to ours found that the specific richness of a grassland included 38 plant species in the control variant and that the natural grasslands have a moderate floristic biodiversity, and by applying the treatments, the specific richness will be reduced simultaneously with the dose of manure applied, results also confirmed by our studies [37]. Another study conducted in 2007, in the context of long-term experiments in the Czech Republic, found that the application of low-dose manure has significantly contributed to improving and maintaining the number of plant species in the vegetal cover [38]. This aspect is also confirmed by our results in the variant with 10 t ha^−1^ manure, where there was a minor change in the floristic composition. At the same time, other authors confirmed that with the application of treatment with N_50_ P_25_K_25_ (V4, in the case of our study), the number of plant species decreased by only four species [39]. These results are similar to those of our research. Although the nitrogen doses are approximately equivalent to the application of the treatment with 10 t ha^−1^ manure (V2) compared to N_50_P_25_K_25_ (V4), the changes in the floristic composition are different, a situation that is due to the stronger effect of mineral fertilizers, which cause more extensive changes and with a high degree of differentiation in the vegetation. The effect of manure on grass depends on several factors. In some research, the external factors taken into account were the weather conditions, the characteristics of the manure, the type of soil and the moisture content of the soil, and the height of the grass [40], a topic that may be worth exploring in future research. In the case of our research, the number of plant species in this variant was reduced by seven. As the fertilizer doses increase, especially with the application of treatments with N_100_P_50_K_50_ and N_150_P_75_K_75_, plant biodiversity is drastically reduced [41,42]. In the case of our research on the application of V5, there was a drastic decrease in the floristic composition of grasslands with HNV, with the phytocenosis only having 25 plant species in the floristic composition. The results of many specialized studies show that the specific richness of HNV grasslands will be reduced at the same time as the applied fertilizer dose [43,44,45], and similar results are confirmed in our studies. At the same time, numerous studies have shown a positive relationship between biodiversity and low-dose fertilization [2,41,45,46]. 

Plant species with indicator value are those that offer valuable information for the researcher on the environmental conditions, the application of maintenance works, and the means of use, the level of anthropogenic influence, etc. [17]. For example, indicator plant species may be particularly useful for HNV grasslands, for which a clear phytodiversity assessment and appropriate practical management must be established [46,47,48]. In our experience, the application of treatments resulted in clear evidence of phytocenoses and a higher number of plant species with indicative value for control phytocenosis. The highest indicator value (100) in our experience was found for the following species: *A. intermedium*, *A. elatius*, *F. arundinacea*, *B. inermis*, *B. secalinus*, *C. stoebe*, *C. lanatus*, *B. falcatum*, *A. angulosum*, *N. arvensis*, and *S. ochroleuca*. However, these plant species of indicative value may be considered as bioindicators for the control, only in the participation registered in the case of control phytocenoses. Our results regarding the identification of plant species with indicator value are also confirmed by other specialized studies, such as [11,17,29,46,49,50]. 

Agronomic factors bring us additional information, useful in explaining the phenomena recorded in the vegetation cover. These factors are essential for establishing the agronomic value and developing appropriate maintenance and use methods for the identified phytocenoses. In this research, we aimed to identify a balance between productivity and biodiversity—in other words, the appropriate dose of input applied so that the biodiversity of the grasslands does not register major changes, and to register an important increase in fodder production for the semi-natural grasslands in our study area. In our experience, as expected, the harvest is favored by the application of organic and mineral fertilization. Dry matter biomass increases as fertilizer doses increase. Significant biomass increases were also obtained by other researchers in this field [51,52,53,54,55,56]. Following this study, the use of fertilizers in moderate doses, namely the application of a 10 t/ha^−1^ manure or N_50_P_25_K_25_, will provide an increase in biomass and exert a minor influence on the diversity of grasslands, being close to the traditional use of grasslands in the Transylvanian Plain (our study area). At the same time, it can be seen in our work that the application of fertilizers (organic and mineral) can cause different biomass crops within the same type of grass—in our case, the type of grass being *F. rupicola*. This method of analysis provides us with valuable information regarding the evaluation of grassland phytocenoses. Such aspects of grassland ecosystems are also confirmed by other researchers in the field [2,57,58,59]. 

## 4. Materials and Methods

### 4.1. Study Site

**Figure 5:**
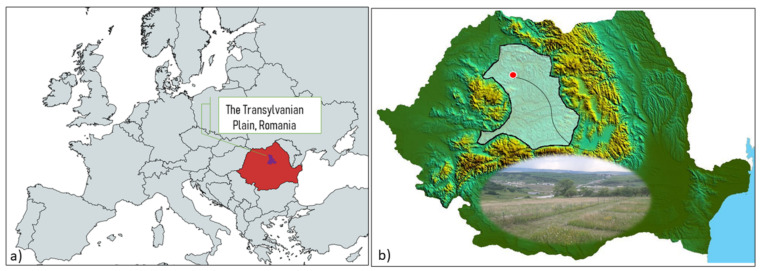
Map of European Union’s NUTS 3 subdivisions (https://mapchart.net/index.html) (accessed on 10 March 2022); (**a**) Map of Romania at the European level; (**b**) Location of studies in the Transylvanian Plain, Romania.

The Transylvanian Depression is famous for its extensive grasslands of various types, most of which have traditionally been used, until now, being mowed by hand or grazed extensively [60]. This natural heritage is now facing changes in use in the form of increased use or abandonment of grasslands, all of which threaten the biodiversity of grasslands with HNV [25]. In Transylvania, there are extensive grasslands with HNV whose biodiversity is remarkable even on a global scale [1,61,62,63]. The Transylvanian Plateau, sometimes called the Transylvanian Basin (45°40′–47°50′ N and 23°00′−25°40′ E [64], Figure 5 [65]), is a hilly area in Central Romania. It is almost entirely surrounded by the Carpathian Mountains, and its altitude varies from around 200 to 700 m.

From the point of view of zoning and vegetation layers in Romania, the Transylvanian Plain is part of the nemoral area [66], with altitudes between 250 and 400 m, usually with clay soils, brown clay, alluvial, and gray. The type of grassland representative of the area is *F. rupicola*, often found on mesoxerophilic biotopes [4]. The productivity of *F. rupicola* grassland is low–medium, with the production of 3.5–6 t per ha green mass and a grazing capacity of 0.4–0.6 LU per ha [67]. Data on the meteorological situation were collected from the weather station located near our experimental field. This research presents meteorological data for 4 experimental years. The highest average was recorded in 2019 (11.4 °C; Table 4, with the lowest average in 2021. It can be seen from the table below that the closest value of the average annual temperature—the average for 60 years—occurred in 2021. Recently, we have been faced with climate change, which has major influences on grassland ecosystems [68,69,70]. 

Regarding the data recorded for precipitation, they are presented as follows: the amount, the average for the last 60 years, and a characterization of the precipitation obtained. It can be seen in Table 4 that the highest rainfall was observed in 2019 and 2020, when 606.0 mm was recorded for each year. According to the climatic characterization, it was found that there were two rainy years. For the year 2018, in terms of rainfall, there was 540.7 mm, with a deviation of +9.7 mm from the annual average for the last 60 years. In 2021, the lowest rainfall was recorded (530.0 mm), with −1 mm average over the last 60 years being 531 mm (Table 4). Table 5 shows the total rainfall and rainfall distribution (mm) for the four experimental years and the long-term total rainfall.

### 4.2. Experimental Set-Up

**Figure 6:**
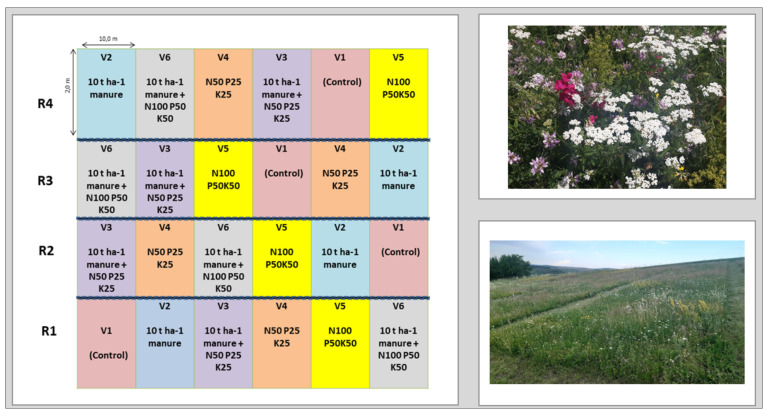
Experimental protocol with organic and mineral fertilizers; V—the fertilization variant; R—repetition. The first image is from the optimal moment when the floristic studies were carried out; the second image shows the arrangement of the experimental variants.

In order to achieve the objectives of this research, an experiment was initialized in 2018. Our experiment was carried out for 4 years (2018–2021). Fertilization of experimental variants was performed in each experimental year (i.e., spring 2018, 2019, 2020, and 2021). Both mineral fertilizers and manure were applied annually. The experiment was devised according to the method of randomized blocks, in four repetitions (blocks), with 6 experimental variants. The experimental plot area totaled 20 m^2^ (Figure 6). The experimental variants were as follows: V1—natural grassland (control); V2—10 t/ha^−1^ manure; V3—10 t/ha^−1^ manure + N_50_ P_25_K_2__5_; V4—N_50_ P_25_K_25_; V5—N_100_ P_50_K_50_; V6—10 t/ha^−1^ manure + N_100_ P_50_K_50_ (Figure 6). The biomass was harvested from each experimental variant, with a mower (BCS 630 WS mower). The mowing height was achieved every year at 4 cm above the ground. Biomass harvesting was conducted only once a year at the optimal time of mowing. The experiments were performed on part of the grasslands at the Agricultural Research and Development Station (ARDS), Turda. The experiments were located at an altitude of 398 m (according to data taken with GPS GARMIN GPSMAP 66S), having the following coordinates: 46°35′15.0″ N 23°57′49.3″ E. 

The experiments were performed on a haplic chernozem soil type. In 2017, before the start of the experiments, a description of the soil profile was created, and physical and chemical data were collected, as presented in Table 6. The analyses were performed by the Office of Pedological and Agrochemical Studies from Cluj-Napoca.

### 4.3. Soil Profile Description—Morphological Description

Am 0–28 cm, clay loam, very dark grayish brown, 10YR 3/3—wet; dark brown 10YR 3/4—dry, moderate moist, weakly cemented, granular structure, very fine to medium. 

Am/Ck 28–52 cm, silty clay, dark greyish brown, 10YR 4/2—wet; brown 10YR 4/3—dry, moist weakly cemented, subangular blocky structure, very fine to medium moderate (grade), friable, moderately plastic, moderately hard, moderately sticky.

Ck1 52–86 cm, silty clay, yellowish brown 10YR 5/4—wet; yellowish brown 10YR 5/8—dry, moist, weakly cemented, subangular blocky structure, fine to coarse, moderate (grade).

Ck2 86–120 cm, silty clay, brownish yellow 10YR 6/6—dry; brownish yellow 10YR 6/8—wet, dry, structureless, fine to coarse single grain, friable, slightly plastic, slightly hard, very strongly calcareous.

### 4.4. Fertilizer Inputs Used

When applying the inputs (organic and mineral), the weather conditions and time intervals recommended in the correct fertilizer application guide were taken into account. The application of the inputs was carried out annually, usually in the first week of April, being considered the optimal time of application. Manure was obtained from households in the area, being a well-fermented manure that corresponded to the guidance on the correct application of fertilizers. The chemical composition of manure is: Nitrogen (N mg/L) 2058, Phosphorus (P mg/L) 515, and Potassium (K mg/L) 2058. Mineral fertilization was performed with N (nitrogen), P (phosphorus), and K (potassium), in a ratio of 16:16:16. Fertilizers were applied every year in 2018, 2019, 2020, and 2021. The application of manure and mineral fertilizers was performed in early spring as follows: 4 April 2018, 31 March 2019, 3 April 2020, and 5 April 2021.

### 4.5. Floristic Studies

Various grassland vegetation research methods are used in the study of grassland systems. Floristic studies were conducted using phyto-population indices: presence/absence, abundance, density, coverage (dominance), abundance–dominance, and frequency [71,72]. Floristic studies were performed using the Braun–Blanquet Abundance–Dominance Assessment Scheme, using three sub-notes [2,66,73]. The floristic determinations were realized every year when the *Poaceae* were in the phenophase of flowering. In our research, we analyzed the floristic data from 2019, 2020, and 2021. The floristic studies were carried out annually. In the experimental area, a mixed management strategy was utilized (mowing and grazing).

### 4.6. Statistical Methods Used

PC-ORD software version 7 was used to process the floristic data obtained in the experimental field (www.pcord.com) (accessed on 15 July 2022) [74], Table 7. For processing, the data obtained were entered in the form of two matrices. In the first matrix, the data on vegetation were introduced, and in the second, the experimental variants were codified. The grouping of the surveys with the numerical analysis of the classification of the experimental data of the present research was carried out with cluster analysis (*Cluster analysis*), where we chose the Euclidean distance index (*Pythagorean*). Ordering floristic plots (PcoA) is a method of data exploration, following which hypotheses can be made about the ecological or agronomic gradients responsible for the variation in the floristic composition of different phytocenoses [66,73]. The measurement of the floristic distance was performed with the help of the similarity index *Sorensen (Bray and Curtis)*. The *Sorensen* distance, measured as percent dissimilarity (PD), is a proportion coefficient measured in city-block space. Sorensen’s Index is very similar to the Jaccard measure and was first used by Czekanowski in 1913 and discovered anew by Sorensen (1948). This index is least affected by large differences in the specific richness, dominance, and total abundance of the species in the areas and sample analyzed [31]. The analysis of plant species with indicator value highlights which species are responsible for differentiating groups. In our research, we performed the analysis of indicator plant species (*Indicator Species Analysis—ISA*) according to the method of *DUFRENE and LEGENDRE* [32]. This method is based on the calculation of the average abundance–dominance (*AD_m_*) and constancy (*K*) of a plant species in all groups. The method combines information on the concentration of species abundance in a particular group and the faithfulness of occurrence of a species in a particular group. It produces indicator values for each species in each group. These are tested for statistical significance using a randomization technique. The method assumes that two or more a priori groups of sample units exist, and that species abundances have been recorded in each of the sample units. The product of these phytopopulational indices will be reported at 100 and will result in the indicative value of the plant species. This indicator value (*INDVAL*) can be between 0 (no indicator value) and 100 (perfect indicator value) [2,11,27,32]. Vegetation traits were calculated as three spectra: naturality—number of species (Spp. no.); Shannon Index (Shannon); ecologic—trophicity (N); soil reaction (R); humidity (U) and agronomic—mowing (C); grazing (P); crushing (S); forage value (VF); yield (Y). For a complete and unified analysis of the three spectra, we use the term agroecological spectrum. Floristic data processing was performed with PC-ORD, version 7, which uses the multivariate analysis of botanical data. Vegetation was quantitatively analyzed with the ANOVA test.

Sorensen indices. The first step is to redefine the traditional binary counts as follows:

S_1_ = total number of species in sample 1;
S_2_ = total number of species in sample 2;
S_12_ = number of species present in both samples;
a = S_12_;
b = S_1_ − S_12_;
c = S_2_ − S_12_.

## 5. Conclusions

The application of inputs on the grasslands of *F. rupicola* determines changes in the composition, which result in a change in dominance and co-dominance between plant species and the installation of new types of grasslands. Each amount of fertilizer applied, organic or mineral, determines a particular floristic composition. At the same time, fertilization strongly influences the participation of species, causing the disappearance or appearance of new plant species. A significant change in the floristic composition occurs when applying mineral fertilizers in moderate to large quantities (N_100_P_50_K_50_). 

The application of fertilizers in moderate doses of 10 t ha^−1^ manure or N_50_P_25_K_25_ does not bring about major changes in the floristic composition and does not endanger the biodiversity of grasslands with HNV, but at the same time, it causes an increase in biomass. The phytocenosis of the control had, in the floristic composition, 12 species of valuable plants. Following the application of inputs (organic and mineral) when applying 10 t ha^−1^ manure, we identified five species of plants with indicator value. The treatment with 10 t ha^−1^ manure + N_50_ P_25_ K_25_ (T3) revealed nine plant species with indicator value. T5 with N_100_ P_50_K_50_ revealed eight species with indicator value and T6 (10 t ha^−1^ manure + N_100_ P_50_K_50_) had, in the floristic composition, a total of seven plant species with indicator value.

## Figures and Tables

**Table 1:** Correlation of experimental factors (vectors) with the ordering axes.

Experimental Factors	Axis 1		Axis 2	
	r	Significance	r	Significance
V1 (control)	0.390	-	0.499	*
V2 10 t ha^−1^ manure	0.262	ns	0.379	ns
V3 10 t/ha^−1^ manure + N_50_ P_25_ K_25_	0.572	**	−0.738	***
V4 N_50_ P_25_ K_25_	−0.265	ns	0.242	ns
V5 N_100_ P_50_K_50_	−0.476	*	−0.140	ns
V6 10 t ha^−1^ manure + N_100_ P_50_ K_50_	−0.446	*	−0.224	ns
Axis importance		78.8%		11.9%

Note: r—correlation coefficient between ordination distances and original distances in n-dimensional space; V1—natural grasslands (control); V2—10 t/ha^−1^ manure; V3—10 t ha^−1^ manure + N_50_ P_25_K_25_; V4—N_50_ P_25_K_25_; V5—N_100_ P_50_K_50_; V6—10/t ha^−1^ manure + N_100_ P_50_K_50_. Significance: *p* ˂ 0.001 ***; *p* ˂ 0.01 **; *p* ˂ 0.05 *; ns—not significant.

**Table 2:** Plant species correlation with the ordination axis in fertilized grassland.

Species	Axis 1				Axis 2			
	*r*	*r*−*sq*	*tau*	*Signif.*	*r*	*r*−*sq*	*tau*	*Signif.*
*Achillea millefolium* L.	−0.662	0.439	−0.550	***	0.342	0.117	0.161	ns
*Agrimonia eupatoria* L.	0.561	0.315	0.325	**	−0.758	0.575	−0.520	***
*Agropyron intermedium* (Host) P. Beauv.	0.390	0.152	0.317	*	0.499	0.249	0.472	**
*Agrostis capillaris* L.	−0.432	0.186	−0.301	*	−0.478	0.229	−0.478	**
*Allium angulosum* L.	0.390	0.152	0.317	*	0.499	0.249	0.472	*
*Arrhenatherum elatius* L.	0.426	0.182	0.475	*	−0.459	0.211	−0.490	**
*Brachypodium pinnatum* (L.) P. Beauv.	0.099	0.010	0.113	ns	0.586	0.343	0.486	**
*Bromus inermis* Leyss.	−0.514	0.264	−0.414	**	−0.158	0.025	−0.190	ns
*Bromus secalinus* L.	0.572	0.327	0.482	**	−0.738	0.544	−0.522	***
*Bromus squarrosus* L.	0.562	0.316	0.428	**	−0.014	0.000	0.079	ns
*Bupleurum falcatum* L.	0.390	0.152	0.317	*	0.499	0.249	0.472	*
*Carex humilis* L.	0.570	0.325	0.631	**	−0.510	0.260	−0.089	**
*Carthamus lanatus* L.	0.390	0.152	0.317	ns	0.499	0.249	0.472	*
*Centaurea stoebe* L.	0.319	0.102	0.323	ns	0.330	0.109	0.460	ns
*Cerastium holosteoides* Fr.	0.516	0.266	0.374	**	0.694	0.482	0.642	***
*Cichorium intybus* L.	0.199	0.039	0.191	ns	0.102	0.010	0.060	ns
*Convolvulus arvensis* L.	−0.251	0.063	−0.053	ns	−0.177	0.031	−0.196	ns
*Coronilla varia* L.	0.021	0.000	−0.023	ns	0.476	0.226	0.523	*
*Dactylis glomerata* L. s. str.	−0.595	0.354	−0.462	**	−0.434	0.188	−0.521	**
*Elymus elongatus* L.	−0.408	0.167	−0.393	*	−0.276	0.076	−0.526	ns
*Elymus repens* (L.) Gould s. str.	0.322	0.104	0.340	ns	0.108	0.012	0.211	ns
*Eryngium campestre* L.	0.395	0.156	0.463	*	−0.074	0.005	−0.118	ns
*Euphorbia cyparissias* L.	−0.422	0.179	−0.449	*	0.114	0.013	−0.002	ns
*Festuca arundinacea* Schreb.	0.572	0.327	0.482	**	−0.738	0.544	−0.522	***
*Festuca pratensis* Huds. s. l.	−0.260	0.068	−0.310	ns	−0.551	0.303	−0.597	**
*Festuca rupicola* Heuff.	−0.967	0.936	−0.842	***	−0.178	0.032	−0.273	ns
*Festuca valesiaca* Schleich. ex. Gaudin s. l.	0.336	0.113	0.317	*	0.250	0.063	0.194	ns
*Fragaria viridis* L.	−0.227	0.051	0.124	ns	−0.047	0.002	0.108	ns
*Galium verum* L. s. str.	−0.150	0.022	0.106	ns	−0.073	0.005	0.067	ns
*Linum catharticum* L.	0.446	0.199	0.367	*	0.224	0.050	0.243	ns
*Lolium perenne* L.	−0.700	0.490	−0.581	***	−0.276	0.076	−0.408	ns
*Lotus corniculatus* L.	−0.283	0.080	−0.296	ns	0.252	0.063	0.181	ns
*Medicago lupulina* L.	−0.364	0.132	−0.339	*	0.404	0.163	0.286	*
*Medicago sativa* L. s. l.	0.177	0.031	−0.235	ns	−0.664	0.441	−0.698	***
*Nigella arvensis* L.	0.390	0.152	0.317	*	0.499	0.249	0.472	*
*Onobrychis viciifolia* Scop.	0.579	0.335	0.539	**	−0.708	0.501	−0.182	***
*Plantago lanceolata* L.	−0.032	0.001	0.097	ns	0.023	0.001	0.064	ns
*Plantago media* L.	−0.616	0.379	−0.465	***	0.066	0.004	−0.037	ns
*Poa angustifolia* L.	0.516	0.266	0.374	**	0.694	0.482	0.642	***
*Poa pratensis* L. s. str.	−0.506	0.256	−0.418	**	−0.200	0.040	−0.417	ns
*Salvia pratensis* L.	0.585	0.342	0.636	**	−0.449	0.202	−0.074	ns
*Scabiosa ochroleuca* L.	0.390	0.152	0.317	*	0.499	0.249	0.472	*
*Trifolium pratense* L.	−0.163	0.027	−0.283	ns	−0.003	0.000	−0.247	ns
*Trifolium repens* L.	0.234	0.055	0.094	ns	−0.160	0.026	−0.203	ns
*Vicia cracca* L. s. str.	0.659	0.435	0.505	***	−0.283	0.080	−0.143	ns
*Viola tricolor* L.	0.440	0.193	0.480	*	−0.195	0.038	−0.136	ns

*r*—correlation coefficient; *r*–*q*—determination coefficient; *tau*—rank (Kendall’s tau) relationships between the ordination. Scores and the individual variables; Significance: *p* ˂ 0.001 ***; *p* ˂ 0.01 **; *p* ˂ 0.05 *; ns—not significant.

**Table 3:** Species with indicator value identified for the treatments applied.

Species	Variant	INDVAL	Mean	Std. Dev.	Signif
*Achillea millefolium* L.	6	28.8	20.7	1.61	0.0002
*Agrimonia eupatoria* L.	3	57.1	24.8	3.37	0.0002
*Agropyron intermedium* (Host) P. Beauv.	1	100.0	10.1	4.32	0.0002
*Agrostis capillaris* L.	6	41.1	22.7	2.44	0.0002
*Allium angulosum* L.	1	100.0	10.1	4.32	0.0002
*Arrhenatherum elatius* L.	3	100.0	11.3	5.07	0.0002
*Brachypodium pinnatum* (L.) P. Beauv.	1	50.0	13.3	3.91	0.0002
*Bromus inermis* Leyss.	5	100.0	10.1	4.30	0.0002
*Bromus secalinus* L.	3	100.0	10.0	4.24	0.0002
*Bromus squarrosus* L.	1	25.0	17.5	2.76	0.0330
*Bupleurum falcatum* L.	1	100.0	10.1	4.32	0.0002
*Carex humilis* L.	3	82.9	14.8	4.96	0.0002
*Carthamus lanatus* L.	1	100.0	10.1	4.32	0.0002
*Centaurea stoebe* L.	1	100.0	11.5	5.09	0.0002
*Cerastium holosteoides* Fr.	1	50.0	13.3	4.01	0.0002
*Convolvulus arvensis* L.	5	43.8	19.6	3.88	0.0002
*Coronilla varia* L.	2	38.9	19.8	4.02	0.0006
*Dactylis glomerata* L. s. str.	6	47.9	21.2	3.04	0.0002
*Elymus elongatus* L.	6	49.0	20.6	4.41	0.0002
*Elymus repens* (L.) Gould s. str.	2	30.0	21.3	3.35	0.0278
*Eryngium campestre* L.	2	60.0	14.9	5.09	0.0002
*Euphorbia cyparissias* L.	5	34.1	23.2	2.66	0.0006
*Festuca arundinacea* Schreb.	3	100.0	10.0	4.24	0.0002
*Festuca pratensis* Huds. s. l.	5	33.7	19.2	3.65	0.0020
*Festuca rupicola* Heuff.	5	24.4	18.9	0.92	0.0002
*Festuca valesiaca* Schleich. ex. Gaudin s. l.	1	46.8	25.3	3.61	0.0002
*Fragaria viridis* L.	5	42.9	21.3	3.21	0.0002
*Galium verum* L. s. str.	5	41.0	22.4	3.75	0.0004
*Lolium perenne* L.	6	44.3	21.9	3.38	0.0002
*Lotus corniculatus* L.	6	25.3	21.1	1.76	0.0250
*Medicago sativa* L. s. l.	3	50.7	20.9	4.51	0.0002
*Nigella arvensis* L.	1	100.0	10.1	4.32	0.0002
*Onobrychis viciifolia* Scop.	3	68.3	23.9	4.40	0.0002
*Plantago lanceolata* L.	5	29.4	20.6	2.77	0.0054
*Poa angustifolia* L.	1	50.0	13.3	4.01	0.0002
*Poa pratensis* L. s. str	4	34.0	19.5	3.79	0.0016
*Salvia pratensis* L.	3	75.0	14.6	4.70	0.0002
*Scabiosa ochroleuca* L.	1	100.0	10.1	4.32	0.0002
*Tragopogon dubius* Scop.	1	33.3	15.7	3.56	0.0012
*Trifolium pratense* L.	6	37.4	22.2	3.61	0.0018
*Trifolium repens* L.	2	29.2	20.7	2.86	0.0092
*Vicia cracca* L. s. str.	2	50.0	13.3	4.02	0.0002
*Viola tricolor* L	3	54.5	14.8	4.95	0.0002

V—variant; INDVAL—indicator value; Std. Dev.—standard deviation; V1—control; V2—10 t ha^−1^ manure; V3—10 t ha^−1^ manure + N_50_ P_25_ K_25_; V4—N_50_ P_25_ K_25_; V5—N_100_ P_50_K_50_; V6—10 t/ha^−1^ manure + N_100_ P_50_K_50_; Significance: *p* ˂ 0.001; *p* ˂ 0.01; *p* ˂ 0.05; ns—not significant.

**Table 4:** Average air temperature for the four experimental years and the long-term average temperature.

Temperature Average Air (°C)	Annual Average			
	2018	2019	2020	2021
Annual temperature	11.2	11.4	10.5	9.9
Average for the last 60 years	9.1	9.1	9.1	9.1
Deviation	+2.1	+2.3	+1.4	+0.8
Characterization	warm	warm	warm	warm

Turda meteorological station (longitude: 23°47′; latitude 46°35′; altitude 427 m).

**Table 5:** Total rainfall and rainfall distribution (mm) for the four experimental years and the long-time term total rainfall.

Rainfall (mm)	Annual Amount			
	2018	2019	2020	2021
Annual amount	540.7	606.0	606.0	530.0
Average for the last 60 years	531.0	531.0	531.0	531.0
Deviation	+9.7	+75.0	+75.0	−1.0
Characterization	normal	rainy	rainy	normal

Turda meteorological station (longitude: 23°47′; latitude 46°35′; altitude 427 m).

**Table 6:** Physical and chemical data from experimental area.

Horizons	Amp	Am/Ck	Ck1	Ck2
Deep (cm)	0–28	28–52	52–86	86–120
Texture				
Coarse sand (2.0–0.2 mm) %	0.73	0.72	0.63	0.34
Fine sand (0.2–0.02 mm)	14.90	19.98	17.92	16.27
Dust I (0.02–0.05 mm) %	9.15	8.78	8.94	9.87
Dust II (0.05–0.002 mm) %	19.15	14.56	20.64	24.14
Clay (<0.002 mm) %	56.07	55.96	51.87	49.38
Texture	SIC	SIC	SIC	SIC
Physical analysis				
Coarse fragments (skeleton) %	-	-	-	-
Bulk density g/cm^3^	1.13	-	1.41	-
Total porosity %	58	-	48	-
Physical analysis				
pH	7.87	7.91	8.19	8.20
Interpretation	Slightly alkaline	Slightly alkaline	Slightly alkaline	Slightly alkaline
Carbonates %	0.7	8.4	24.0	32.6
Humus %	3.49	2.89	-	-
N total %	0.207	0.148	-	-
P mobile (ppm)	65	20	-	-
K mobile (ppm)	400	332	-	-

Physical and chemical data—Office of Pedological and Agrochemical Studies from Cluj-Napoca; Soil samples were taken from the experimental research field.

**Table 7:** Reformulation of the Jaccard and Sorensen indices for presence–absence data and abundance data [74].

**Index**	**Presence–Absence Based on a, b, c**	Presence–Absence Based on S_1_, S_2_, S_12_	Abundance Based (See Definitions Below)
Sorensen	$\frac{a}{a+b+c}$	$\frac{S_{12}}{S_{1}+S_{2}-S_{12}}$	$\frac{UV}{U+V-UV}$
Jaccard	$\frac{2a}{\left(2a+b+c\right)}$	$\frac{2S_{12}}{S_{1}+S_{2}}$	$\frac{2UV}{U+V}$

## Data Availability

Not applicable.
